# The Prevalence of Abnormal Glucose Metabolism and Its Correlates in the Zhuang Nationality, Nanning, Guangxi Province

**DOI:** 10.1002/jcla.70117

**Published:** 2025-10-09

**Authors:** Qin Yuanyuan, Liao Lin, Chen Qingyun, Huang Yunhua, Lin Faquan

**Affiliations:** ^1^ Key Laboratory of Clinical Laboratory Medicine of Guangxi Department of Education, Department of Clinical Laboratory The First Affiliated Hospital of Guangxi Medical University Nanning China; ^2^ Health Management Department The First Affiliated Hospital of Guangxi Medical University Nanning China

**Keywords:** abnormal glucose metabolism, influencing factors, pre‐diabetes, prevalence, type 2 diabetes mellitus

## Abstract

**Background:**

The prevalence of diabetes prevalence has surged in China, becoming a severe public health issue. However, data on abnormal glucose metabolism among ethnic minorities, particularly the Zhuang population, remains limited. This study aims to fill this gap by analyzing the prevalence and risk factors of glucose abnormalities in the Zhuang population.

**Methods:**

In 2017, data were gathered from a sample of 1957 Zhuang adults, aged 18 and older, who underwent a comprehensive physical examination at the First Affiliated Hospital of Guangxi Medical University. Variables included demographic information, lifestyle habits, anthropometric measurements, and biochemical indicators. Abnormal glucose metabolism was diagnosed based on fasting blood glucose (GLU) and 2‐h postprandial glucose (2hPG) levels. Statistical analysis was performed using SPSS 24.0.

**Results:**

This study shows reveals a 37.52% prevalence of abnormal glucose metabolism among the Zhuang population in Nanning, with Type 2 diabetes mellitus (T2DM) and pre‐diabetes (PDM) rates of 9.7% and 23.17%, respectively. Risk factors identified include age, high BMI, hypertension, dyslipidemia, and alcohol consumption.

**Conclusion:**

This study highlights that the prevalence of abnormal glucose metabolism is very high in Nanning, Guangxi. Targeted intervention measures, such as enhanced health education, should be implemented for high‐risk populations to effectively implement diabetes prevention policies and interventions.

**Trial Registration:**

This study is not a clinical trial, so there is no clinical trial registry

## Graphical abstract

Prevalence of T2DM and PDM in different age groups; * was considered statistically significant. They were divided into three groups according to age: 40 years old or younger, 41–60 years old, and more than 60 years old. The prevalence of T2DM and PDM was statistically significant in different age groups.

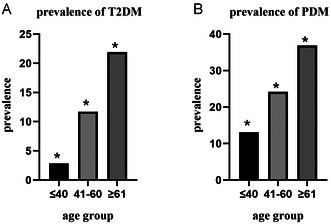

## 1 Introduction

The prevalence of diabetes has soared in China, emerging as a severe public health issue with the country's rapid economic development, the improvement of people's living standards, and the intensification of population aging. According to the 10th edition of the International Diabetes Federation (IDF) Global Diabetes Report, China had approximately 141 million adults aged between 20 and 79 years with diabetes in 2021, the highest number globally, with an age‐adjusted prevalence of 10.6%. T2DM is characterized by high incidence rates, numerous complications, and elevated mortality rates. Due to its escalating medical burden, T2DM has become a significant concern in China [1]. However, existing studies predominantly focus on Han Chinese populations, with limited data on ethnic minorities, particularly the Zhuang nationality. In contrast, our study is the first to systematically analyze abnormal glucose metabolism in the Zhuang population of Nanning, Guangxi, using a large‐scale physical examination cohort.

PDM represents an intermediate stage of abnormal glucose metabolism and is a precursor to T2DM [2], encompassing impaired fasting glucose (IFG), impaired glucose tolerance (IGT), and a mixed state of both (IFG + IGT), and represents an intermediate hyperglycemic state between normal blood glucose levels and diabetes. Abnormal glucose metabolism encompasses a spectrum of disorders, including diabetes (DM) and PDM. Although the latter is recognized as an intermediate stage between normal blood glucose levels and the former, it carries an annual risk of 2%–10% of progressing to DM. The IDF predicts that, by 2045, 700 million people will be living with DM, and 548 million will have developed IGT. Studies have shown that individuals in the PDM stage face a higher risk of chronic kidney and cardiovascular disease, and mortality compared to those with normal blood glucose levels [3, 4]. Given the associated risks of elevated blood glucose levels in PDM, controlling blood glucose is crucial for preventing T2DM and micro‐ and macrovascular diseases [5]. Blood glucose monitoring, as a vital component of DM management, has become an indispensable tool in the prevention and control of DM. The Zhuang nationality, constituting the largest ethnic minority populace in China, predominantly resides in the Guangxi Zhuang Autonomous Region and exhibits distinctive dietary patterns and lifestyle characteristics. Diabetes prevalence among ethnic minorities exhibits distinct geographic and dietary patterns [6]. This study aims to explore the prevalence of abnormal glucose metabolism and its risk factors among the Zhuang population in Nanning, which may offer insights for localized prevention and intervention strategies for glucose‐related disorders in this specific subgroup.

## 2 Patients and Methods

### 2.1 Patients

This study utilized employed a population‐based, stratified multistage random sampling approach to ensure representativeness. Of 1997 eligible individuals, 1951 completed the study (response rate: 97.7%) at the First Affiliated Hospital of Guangxi Medical University. Inclusion criteria included being a Zhuang ethnic member aged 18 and above with having a long‐term history of residence or work history in Nanning. For clarity, Zhuang ethnicity was defined by the nationality listed on the Chinese resident identity card, with all direct ancestors across three generations belonging to the Zhuang ethnicity. Participants with acute illnesses, incomplete records, or unwillingness to participate were excluded.

### 2.2 Methods

#### 2.2.1 Detection Methods

For the survey, we distributed administered structured questionnaires that comprehensively covered demographics, lifestyle factors, personal medical histories, histories of chronic illnesses, medication use, and other relevant pertinent details. Standardized measurement protocols were adopted implemented to ensure consistency in assessing height, weight, waist circumference, and blood pressure across all participants. In the morning, fasting blood samples were collected to assess serum GLU, lipids (including total cholesterol (TC), triglycerides (TG), high‐density lipoprotein cholesterol (HDL‐C), and low‐density lipoprotein cholesterol (LDL‐C)), urea nitrogen (BUN), creatinine, and uric acid (UA). All participants underwent an oral glucose tolerance test, which involved consuming a standardized dose of 75 g of anhydrous glucose powder dissolved in a 200–300 mL solution of warm water. Subsequently, their blood glucose levels were measured at the 2hPG. Specifically, GLU and 2hPG levels were assayed using the oxidase‐peroxidase method. Additionally, TC was quantified through the cholesterol oxidase method, TG was evaluated using the glycerol phosphate oxidase method, HDL‐C and LDL‐C were assessed using direct methods, BUN was detected via the kinetic rate method, creatinine was measured by the sarcosine oxidase method, and UA was determined using the uricase method.

#### 2.2.2 Diagnostic Criteria

Abnormal glucose metabolism encompasses T2DM and PDM. The specific criteria are as follows: T2DM is diagnosed when FPG is ≥ 7.0 mmol/L and/or 2hPG is ≥ 11.1 mmol/L; IFG is diagnosed when FPG is between 6.1 and 7.0 mmol/L and 2hPG is < 7.8 mmol/L; IGT is diagnosed when FPG is < 6.1 mmol/L and 2hPG is between 7.8 and 11.1 mmol/L. Individuals meeting both the IFG and IGT criteria are diagnosed as having PDM. Smokers were defined as individuals actively smoking at the time of the survey and who had a cumulative smoking history of at least 100 cigarette ‐ packs [7]. Individuals classified as drinkers were those with a history of alcohol consumption spanning a year and consuming more than 30 g of alcohol weekly. Drawing upon the 2018 Revised Edition of the Chinese Guidelines on Hypertension Prevention and Management, hypertension is defined as a systolic blood pressure of 140 mmHg or higher (1 mmHg = 0.133 kPa) and/or a diastolic blood pressure of 90 mmHg or higher. The Body Mass Index (BMI) formula is: BMI = weight (kg)/height (m)^2^. The study categorized educational attainment into four levels: primary school or below, junior middle school, technical secondary school or high school, and junior college or above. Annual household income was stratified into three brackets: 10,000 yuan or less, 10,001 to 29,999 yuan, and 30,000 yuan or above. Furthermore, high‐fat diets were classified into three degrees: minimal, moderate, and substantial [8]. For vegetable intake, consuming more than 500 g of vegetables per day was considered high, while not consuming any vegetables or consuming less than 300 g per day was considered little, while for fruit intake, consuming more than 1000 g of fruit per day was considered high, while not consuming any fruit or less than 200 g per day was considered little [8].

### 2.3 Statistical Analysis

Statistical analyses were conducted using utilizing IBM SPSS Statistics version 24.0. Continuous variables were expressed as presented as means with standard deviations, whereas categorical variables were described delineated as counts and percentages. Differences in gender, age, smoking, drinking, hypertension, educational level, household income, high‐fat diet, high fruit intake, adequate vegetable intake, and frequency of tea drinking were compared between patients with T2DM, PDM, and healthy individuals normal people using Chi‐square tests. Age, waist circumference, BMI, serum TC, TG, HDL‐C, LDL‐C, GLU, BUN, and creatinine concentrations were compared between patients with T2DM, PDM, and healthy individuals normal people using One‐way ANOVA, followed by Tukey HSD post hoc tests. Independent risk factors for T2DM and PDM were determined by bivariate non‐conditional logistic regression analysis. Bivariate non‐conditional logistic regression was employed to estimate odds ratios (ORs) and 95% confidence intervals (CIs), and a *p*‐value of < 0.05 was deemed indicative of statistical significance.

## 3 Results

### 3.1 Detection of Abnormal Glucose Metabolism in the Study Subjects

**FIGURE 1:**
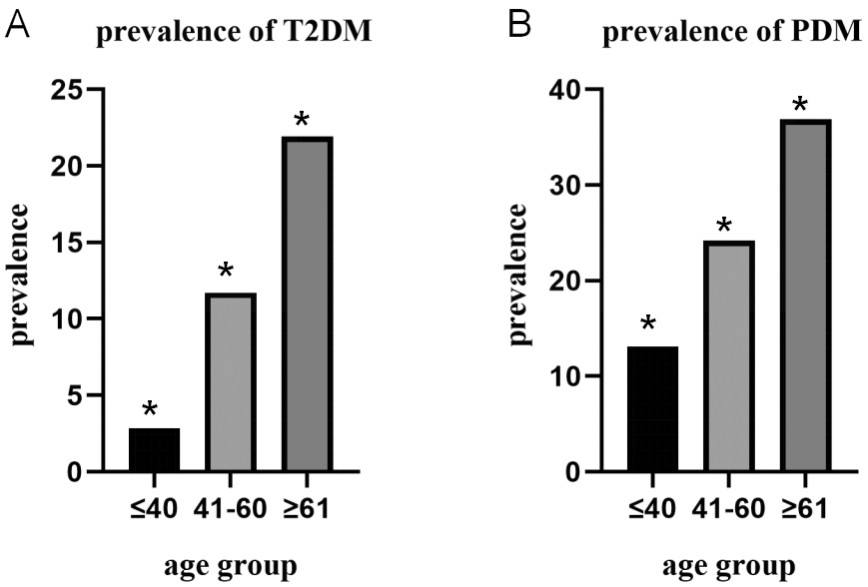
Prevalence of T2DM and PDM in different age groups; * was considered statistically significant.

We collected gathered data from 1951 patients undergoing physical exams, including 732 males (37.5%) and 1219 females (62.5%) who completed the study (response rate: 97.7%). A total of 732 cases of abnormal glucose metabolism were detected in this study, accounting for 37.52% of the study subjects. Among them, T2DM and PDM accounted for 9.7% and 23.17%, respectively. Upon comparison, the detection rates of T2DM and PDM were higher in males than in females (*p* < 0.05), as shown in Table 1. They were divided into three groups according to age: 40 years old or younger, 41–60 years old, and more than 60 years old. The prevalence of T2DM and PDM was statistically significant in different age groups, as shown in Figure 1.

**TABLE 1:** Detection of various types of abnormal glucose metabolism in the overall study population and by gender.

Various types	All (1951)			Males (732)			Females (1219)			*p*
	*n*	%	95% confidence intervals	*n*	%	95% confidence intervals	*n*	%	95% confidence intervals	
Abnormal glucose metabolism	642	37.5	30.8–35.0	256	35	31.50–38.44	386	31.7	29.08–34.26	0.02
T2DM	190	9.7	8.4–11.1	89	12.2	9.79–14.53	101	8.28	6.73–9.83	0.005
PDM	452	23.17	21.3–25.0	167	22.81	19.79–25.83	285	23.38	21.03–25.73	0.824
Normal group	1309	62.5	65.0–69.2	476	65	61.6–68.4	833	68.3	65.7–70.9	

Abbreviations: PDM, pre‐diabetes; T2DM, type 2 diabetes mellitus.

### 3.2 Comparisons of Each Index Between Patients With and Without Abnormal Glucose Metabolism

Age, waist circumference, TC, TG, LDL, UA, GLU, 2hPG, HbA1C, BMI, drinking, hypertension, educational level, and frequency of tea drinking were significantly increased, and HDL was decreased in patients with and without T2DM. Age, waist circumference, TC, TG, LDL, UA, creatinine, GLU, 2hPG, HbA1C, BMI, hypertension, and educational level were significantly increased, and HDL was decreased in patients with and without PDM. Compared with PDM patients, waist circumference, TG, BMI, hypertension, and frequency of tea drinking were significantly increased, and HDL was decreased in T2DM patients, as shown in Table 2.

**TABLE 2:** Comparisons of each index between patients with and without abnormal glucose metabolism.

	T2DM (190)	PDM (452)	Normal group (1309)	*t*/*F*	*p*
Age (year)	56.79 ± 8.947^a^	55.49 ± 10.151^a^	50.06 ± 12.205	56.27	0.000
Waist circumference	85.28 ± 10.56^a^, ^b^	83.61 ± 9.74^a^	79.07 ± 9.376	61.46	0.000
TC (mmol/L)	5.98 ± 1.25^a^	6.00 ± 1.12^a^	5.67 ± 1.113	17.09	0.000
TG (mmol/L)	2.04 ± 1.81^a^, ^b^	1.62 ± 1.25^a^	1.21 ± 1.68	28.15	0.000
LDL (mmol/L)	3.41 ± 0.93^a^	3.46 ± 0.87^a^	3.21 ± 0.87	15.47	0.000
HDL (mmol/L)	1.68 ± 0.48^a^, ^b^	1.77 ± 0.46^a^	1.88 ± 0.46	22.07	0.000
UA (μmol/L)	334.41 ± 93.30^a^	331.02 ± 83.24^a^	309.54 ± 79.64	16.35	0.000
BUN (mmol/L)	4.96 ± 1.38	5.05 ± 1.38	5.003 ± 1.47	0.28	0.754
Creatinine (μmol/L)	69.46 ± 20.25	69.72 ± 16.09^a^	66.94 ± 18.12	4.94	0.007
GLU (mmol/L)	8.23 ± 3.12^a^, ^b^	5.83 ± 0.65^a^	5.20 ± 0.44	660.71	0.000
2hPG (mmol/L)	14.57 ± 5.85^a^, ^b^	8.35 ± 1.71^a^	5.44 ± 1.29	1451.98	0.000
HbA1C (%)	7.09 ± 2.16^a^, ^b^	5.65 ± 0.57^a^	5.32 ± 0.49	378.51	0.000
BMI	25.47 ± 4.31^a^, ^b^	24.75 ± 3.70^a^	23.15 ± 3.37	58.80	0.000
Males [*n*]	101 (53.16)^a^	167 (36.94)	476 (36.36)	21.05	0.000
Smoking [*n*]				1.92	0.383
No	148	372	1071 (81.8)	—	
Yes	42	80	238 (18.5)	—	
Drinking [*n*]				9.13	0.01
No	126^a^	330	997 (68.2)	—	
Yes	64	122	312 (23.8)	—	
Hypertension [*n*]				391.03	0.000
No	57^a^, ^b^	137^a^	718 (54.9)		
Yes	133	315	591 (55.1)		
Educational level [*n*]				12.97	0.011
Primary school or below	63^a^	141^a^	365 (64.1)	—	
Junior high school, technical secondary school or senior high school	117	282	803 (66.8)	—	
University or college education	10	29	141 (2)	—	
Household income [*n*]				5.54	0.236
10,000 and below	63	149	408 (31.2)		
10,000 ~ 30,000	87	237	691 (52.8)		
More than 30,000	40	66	210 (16)		
High fat diet [*n*]				2.59	0.629
Little	48	93	284 (21.7)		
General	118	310	874 (66.8)		
Many	24	49	151 (11.5)		
High fruit intake [*n*]				1.84	0.766
Little	55	126	393 (30)		
General	121	281	794 (60.7)		
Many	14	45	122 (9.3)		
Adequate vegetable intake [*n*]				2.78	0.595
Little	18	28	100 (7.6)		
General	107	269	783 (59.8)		
Many	65	155	426 (32.5)		
Frequency of tea drinking [*n*]				11.86	0.018
Never	86^a^, ^b^	212	663 (40.9)		
Little	75	203	533 (32.9)		
Days	29	37	113 (26.3)		

Abbreviations: 2hPG, 2‐h postprandial glucose; BUN, urea nitrogen; GLU, blood glucose; HDL‐C, high‐density lipoprotein cholesterol; LDL‐C, low‐density lipoprotein cholesterol; TC, total cholesterol; TG, triglycerides; UA, uric acid; BMI,the Body Mass Index.

^a^ Considered statistically significant between patients with T2DM and without a healthy individuals normal group.

^b^ Considered statistically significant between patients with T2DM and PDM.

### 3.3 Abnormal Glucose Metabolism‐Related Risk Factors

Using the presence or absence of PDM and T2DM as dependent variables, respectively, and statistically significant factors from univariate analysis as independent variables, multivariate logistic regression analyses were conducted. The results revealed that age, TC, BMI, hypertension, and drinking were risk factors for T2DM, whereas LDL‐C and HDL‐C were protective factors for T2DM. Similarly, age, heart rate, TC, BMI, and hypertension were identified as risk factors for PDM, and LDL‐C and HDL‐C emerged as protective factors for PDM, as shown in Tables 3 and 4.

**TABLE 3:** Multivariate logistic regression analysis of factors influencing T2DM among the study participants.

	*B*	SE	Wald	*p*	Exp(*B*)	95% CI
Age	0.049	0.01	25.08	0.000	1.051	1.03–1.071
Waist circumference	0.004	0.012	0.116	0.733	1.004	0.980–1.029
TC	0.958	0.269	12.716	0.000	2.606	1.54–4.08
UA	−0.001	0.001	0.375	0.541	0.999	0.997–1.002
LDL‐C	−0.948	0.308	9.468	0.002	0.388	0.212–0.709
HDL‐C	−1.417	0.33	18.45	0.000	0.243	0.127–0.463
TG	−0.097	0.067	2.097	0.148	0.907	0.795–1.035
BMI	0.109	0.032	11.652	0.001	1.115	1.048–1.187
Hypertension	0.403	0.187	4.668	0.031	1.497	1.038–2.159
Educational level	−0.3	0.396	0.574	0.449	0.741	0.341–1.61
Drinking	0.504	0.196	6.579	0.01	1.655	1.126–2.432

Abbreviations: HDL‐C, high‐density lipoprotein cholesterol; LDL‐C, low‐density lipoprotein cholesterol; TC, total cholesterol; TG, triglycerides; UA, uric acid; BMI, the Body Mass Index.

**TABLE 4:** Multivariate logistic regression analysis of factors influencing PDM among the study participants.

	*B*	SE	Wald	*p*	Exp(*B*)	95% CI
Age	0.029	0.007	20.163	0.000	1.03	1.017–1.043
Waist circumference	0.005	0.009	0.322	0.571	1.005	0.987–1.023
TC	0.918	0.226	16.521	0.000	2.503	1.608–3.896
UA	0.001	0.001	1.706	0.192	1.001	0.999–1.003
LDL‐C	−0.856	0.256	11.167	0.001	0.425	0.257–0.702
HDL‐C	−1.163	0.259	20.248	0.000	0.312	0.188–0.519
TG	−0.120	0.063	3.624	0.057	0.887	0.785–1.004
Creatinine	−0.003	0.004	0.801	0.371	0.997	0.989–1.004
BMI	0.065	0.023	7.909	0.005	1.067	1.02–1.116
Hypertension	0.609	0.128	22.462	0.000	1.838	1.429–2.365
Educational level	−0.112	0.265	0.18	0.671	0.894	0.532–1.501

Abbreviations: HDL‐C, high‐density lipoprotein cholesterol; LDL‐C, low‐density lipoprotein cholesterol; TC, total cholesterol; TG, triglycerides; UA, uric acid; BMI, the Body Mass Index.

## 4 Discussion

The prevalence of T2DM and PDM has exhibited shown a sustained upward trend with the development of the national economy, the continuous improvement of people's living standards, the accumulation of unhealthy lifestyles among residents, and the aging of the population [9]. Therefore, investigating the prevalence of T2DM and PDM in Nanning, Guangxi Province, is of great significance for controlling levels of glucose and abnormal glucose metabolism risk factors in the population and for formulating effective prevention and treatment measures.

According to existing literature, the prevalence rate of T2DM among adults in China has risen from less than 3.7% in 1990 to 6.6% in 2016 [10]. The results of this study indicate that the prevalence rate of T2DM among adults in Nanning is 9.7%, which is higher than the survey findings of Guangxi in 2008 (7.1%) [11], and lower than the prevalence rate in Beijing in 2022 (13.9%) [12]. The results also indicate that the prevalence rate of PDM among adults in Nanning is 23.17%, which is significantly lower than the national PDM prevalence rate of 35.70% in 2013; the prevalence of diabetes among the Tibetan and Muslim populations is significantly lower than that among the Han ethnic group [13]. Similarly, nationwide surveys conducted between 2007 and 2017 indicate that the prevalence of PDM in China stands at 35.20%; the prevalence of diabetes varies across different ethnicities. Han ethnicity had the highest prevalence of diabetes (12.8%) and Hui ethnicity had the lowest (6.3%) [14]. Such differences may be due to different geographical environments, demographic backgrounds, and living habits [10, 11, 12, 13, 14].

A survey showed that the prevalence of T2DM is higher in males than in females [15]. Jin et al. [16] also found in a population study conducted in mainland China that the prevalence of T2DM in males was significantly higher than that in females, which is consistent with our findings. Our results suggest that the prevalence of T2DM is higher in males than in females. This discrepancy may be related to physiological hormones in males and females. Androgens may exert an antagonistic effect on insulin action, leading to decreased insulin sensitivity and an elevated risk of developing diabetes [17]. In addition, the difference between males and females may be related to daily living habits, such as males engaging more frequently in entertainment, smoking, and drinking, and experiencing greater work pressure. This difference may also be caused by the differences in diabetes incidence risk between males and females, which may be attributed to a multitude of factors, including biological, lifestyle, and societal considerations [16]. Some studies have shown that the prevalence of PDM is higher in males than in females [18]. However, the findings of this study indicate no statistical difference in the prevalence of PDM between both genders. A possible reason for this may be the changes in modern lifestyles, where both males and females increasingly face issues such as high‐sugar, high‐fat, and high‐calorie diets, coupled with a lack of physical activity. These issues are ubiquitous across both genders and may therefore not exert a significant influence on the prevalence of PDM.

Previous studies have demonstrated that age, BMI, and hypertension are associated with abnormal glucose metabolism [16, 18]. The occurrence of T2DM is closely related to pancreatic islet cells. The mechanism underlying this disease involves inadequate or insufficient insulin action, leading to elevated blood glucose levels in the body, ultimately triggering T2DM. As the body ages, there is a decline in the metabolic rate, accompanied by varying degrees of decline in organ and tissue function, with a particularly notable decline in pancreatic islet cells. This results in insufficient insulin secretion by the body, which cannot meet the body's needs, leading to elevated blood glucose levels and an increased risk of DM development [19, 20]. The inevitable process of aging and degeneration occurs in various organs of the body, leading to an increased risk of developing multiple chronic diseases, including T2DM. Consequently, older age is a high‐risk factor for T2DM. Therefore, enhancing blood glucose monitoring among older residents can facilitate the early detection of abnormal glucose metabolism and allow for timely intervention measures to be implemented.

BMI is used to assess an individual's nutritional status, with a BMI ≥ 25 being classified as overweight. Overweight and obesity are major factors contributing to insulin resistance, which serves as the foundation for chronic diseases, such as DM [21]. In overweight and obese populations, excess adiposity can lead to an increase in lipolysis, resulting in elevated levels of free fatty acids in the bloodstream. This, in turn, impairs the liver's ability to efficiently take up and utilize glucose in an insulin‐mediated manner, ultimately leading to heightened blood glucose levels in the circulation [22]. A higher BMI is an independent risk factor for DM [23], and the level and dynamic changes in BMI are closely associated with DM [24]. Hypertension and T2DM exhibit a mutually reinforcing relationship. If abnormal elevations in blood pressure within the body are not effectively controlled, they can lead to insulin resistance, resulting in abnormal blood glucose levels and increasing the probability of the onset of T2DM. The findings of this study indicate that advanced age, obesity, and hypertension are risk factors for abnormal glucose metabolism, which corroborates the aforementioned viewpoints.

Previous studies have demonstrated an association between dyslipidemia and abnormal glucose metabolism [25]. Several studies have reported that patients with T2DM often exhibit elevated levels of TG, LDL‐C, and very low‐VLDL‐C, along with decreased levels of HDL‐C [26]. Patients with Type 1 diabetes (T1DM) and T2DM who are at high or extremely high cardiovascular risk rarely achieve the target levels for LDL‐C and non‐HDL‐C [27]. Huang et al. [28] conducted a survey among a physically examined population in Nanning and found that the levels of 2hPG and FPG were higher in the dyslipidemia group compared to the non‐dyslipidemia group. Furthermore, an elevated 2hPG level was identified as a risk factor for dyslipidemia. The disordered lipid profile has its pathogenesis in insulin resistance, hyperinsulinemia, and abnormal adipokine levels, all commonly seen in DM [29]. The hyperinsulinemic state of the body enhances hepatic production of VLDL particles, which contribute to a high fasting TG level [30]. The results of this study indicate that the levels of TC, TG, and LDL‐C were higher in the abnormal glucose metabolism group compared to the normal control group, while the level of HDL‐C was lower. Additionally, elevated TC and reduced HDL‐C were identified as risk factors for abnormal glucose metabolism, which corroborates the aforementioned viewpoint.

The findings of this study suggest that alcohol consumption is a risk factor for T2DM. Excessive alcohol consumption is not only an independent risk factor for the development of DM, but also a significant influencing factor on glycemic control among diabetic patients [31]. The mechanism may lie in the fact that excessive alcohol consumption and its metabolites can lead to insulin resistance, thereby affecting cellular glucose utilization. Reports have shown that abnormal glucose metabolism is associated with lifestyle and dietary habits [32]. However, the conclusions of this study are inconsistent with previous research. Possible reasons for this discrepancy may include differences in sample selection between the current study and previous ones, such as variations in sample size, geographical distribution of the samples, age structure, and gender ratio.

This study has several key limitations. It's a cross‐sectional survey conducted exclusively only done in Nanning, Guangxi, so the results may not apply to other regions. Using a hospital‐based sample could cause selection bias. Hospital patients might have different health features and behaviors from the general public, making our sample less representative. The cross‐Self‐funded Scientific Research Project of Guangxi Provincial Health Commissionsectional design means we can't determine figure out if one factor causes changes in another, like whether a lifestyle factor directly leads to high serum glucose. Longitudinal studies are needed to establish causality. Also, the sample size is small, which may affect result reliability. Larger samples are required for more solid conclusions. In short, be cautious when interpreting these results, and future research should address these issues.

In summary, the data from this study indicate that the prevalence of abnormal glucose metabolism is very high in Nanning, Guangxi. Abnormal glucose metabolism can induce metabolic syndrome and cardiocerebrovascular disease, which has become an important public health problem in China. The global burden of PDM is substantial and growing, and enhancing PDM surveillance is necessary to effectively implement DM prevention policies and interventions. Age, high BMI, hypertension, dyslipidemia, and alcohol consumption are risk factors for abnormal glucose metabolism. We propose emphasizing the reinforcement of health education on topics such as healthy eating, regular physical activity, weight management, smoking cessation, and alcohol moderation. Additionally, we can leverage community resources, the family doctor system, and mobile health technologies to enhance the reach and effectiveness of health education. Furthermore, considering the cultural and linguistic specificities of the Zhuang ethnic population, we can also develop health education materials and tools that are culturally appropriate and linguistically accessible to them. Regular blood glucose monitoring should be conducted to detect abnormal glucose metabolism early and implement early interventions, thereby reducing the incidence of T2DM and its complications.

## Author Contributions

Kindly add all authors' names and their contributions to Authors' contributions. Qin Yuanyuan and Liao Lin conceptualized and designed the study, collected and analyzed the data, and wrote the manuscript. Chen Qingyun and Huang Yunhua contributed to the data analysis and interpretation, and critically reviewed the manuscript. Lin Faquan provided technical support and edited the manuscript. All authors read and approved the final manuscript.

## Ethics Statement

This study has received ethical approval from the Zhongda Hospital Southeast University Ethics Committee (Approval Number: 2016ZDSYLL092‐P01).

## Consent

All participants provided verbal consent for the use of their personal information and clinical data in this study. Participants provided consent for the publication of their data in this study.

## Conflicts of Interest

The authors declare no conflicts of interest.

## Data Availability

The datasets and materials used in this study are available from the corresponding author upon reasonable request.
